# Prime Editing in Dividing and Quiescent Cells

**DOI:** 10.3390/ijms26083596

**Published:** 2025-04-11

**Authors:** Irina O. Petrova, Svetlana A. Smirnikhina

**Affiliations:** Laboratory of Genome Editing, Research Center for Medical Genetics, Moskvorechye 1, 115478 Moscow, Russia

**Keywords:** genome editing, cell cycle, DNA replication, mismatch repair

## Abstract

Prime editing is a method of genome editing based on reverse transcription. Recent results have shown its elevated efficiency in dividing cells, which raises some questions regarding the mechanism of this effect, because prime editing does not employ homology-driven repair. This mini review aims to identify the reason for this phenomenon and find a possible solution to the problems that it poses. In dividing cells, prime editing takes advantage of high levels of dNTPs and active endonuclease and ligase machinery, such as FEN1 endonuclease and LIG1 ligase, but DNA mismatch repair, which is closely associated with replication, works against prime editing. Prime editing is a method which relies on retroviral reverse transcription, so mechanisms of intrinsic anti-retroviral defense should also work against editing. One of the factors which drastically reduce the efficiency of reverse translation is SAMHD1, which maintains low levels of dNTPs in non-dividing cells. Recent works aimed at the mitigation of SAMHD1 function demonstrated a significant increase in prime editing efficiency.

## 1. Introduction

Prime editing (PE) is one of the most promising genome editing methods [1]. By utilizing this approach, similarly to other genome editing methods, it becomes feasible to make precise alterations to the genome for the purpose of generating genetically modified organisms. This can be applied in areas such as agriculture [2] or animal disease modeling [3]. Furthermore, it enables the examination of various conditions’ pathogenesis and the advancement of novel gene therapy techniques. The latter aspect holds particular significance presently, owing to advancements in in vivo delivery systems [4]. The PE method enables precise modifications to the genome for therapeutic purposes, distinguishing itself from homology-directed repair (HDR)-based CRISPR-Cas by its specificity and accuracy. Although prime editing technology has yet to be the subject of any clinical trials, we firmly believe that this will occur in the immediate future.

**Figure 1:**
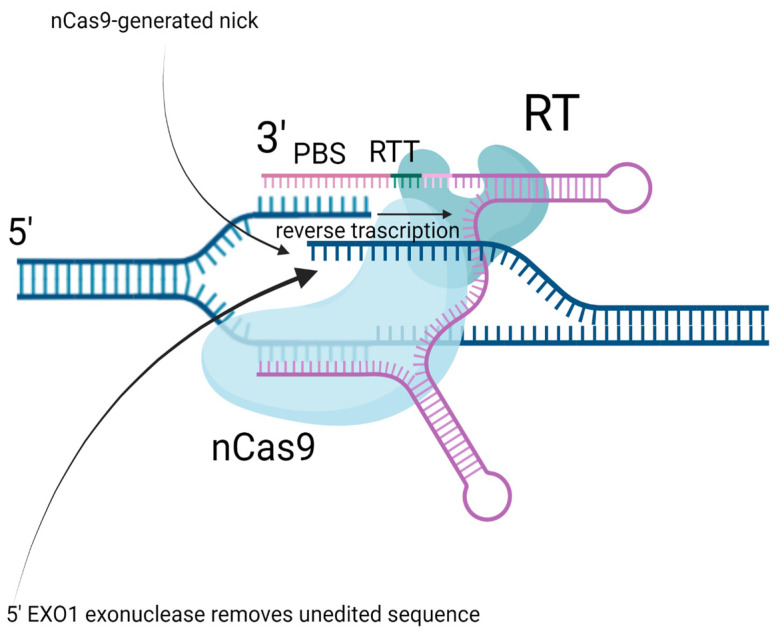
A schematic representation of the prime editing mechanism. The prime editor fusion protein consists of nCas9 nickase and reverse transcriptase (RT). The prime editing guide RNA includes a guide RNA fragment that binds the DNA target to expose the opposite strand, which is nicked by nCas9 (upper arrow). The nicked fragment binds with the primer binding site (PBS) of pegRNA. The nicked strand serves as a primer for reverse transcription, which elongates the nicked strand using a reverse transcription template (RTT) of pegRNA as a matrix. EXO1 exonuclease removes the unedited 5′ flap (lower arrow). Created using Biorender.

Prime editing uses an engineered Moloney murine leukemia virus reverse transcriptase (MMLV RT) that is fused to Cas9 nickase (nCas9) and a single prime editing guide RNA (pegRNA), consisting of a guide RNA (a spacer), a primer binding sequence (PBS) for initiation of reverse transcription, and a reverse transcription template (RTT) sequence carrying the desired edit. nCas9 induces a single-strand break next to the intended edit site, leading to nicked strand hybridization with PBS of pegRNA. After that, the reverse transcriptase synthesizes a new DNA strand using RTT as a matrix. Newly synthesized strand forms a heteroduplex with the non-edited strand, replacing the original nicked strand, whose flapping 5′-terminus is removed by host flap endonuclease 1 (FEN1) or by exonuclease 1 (EXO1) (Figure 1). The final event of the heteroduplex resolution and the edit incorporation (or lack thereof) is dependent on DNA repair mechanisms. DNA repair can either function in favor of editing, removing the non-edited strand, or against it, removing the newly synthesized edited strand. Finally, the nicked strand is repaired by the host DNA ligase.

Compared to CRISPR-Cas, PE has several advantages. First, it demonstrates higher precision by not relying on double-strand breaks. Second, it has a lower off-target effect. Third, it requires a single guide RNA for target recognition and as a matrix for editing. Compared to base editing, it is more flexible, because it does not require different proteins for different types of edits. On the other hand, the increased size of the PE complex compared to regular Cas9 complicates its delivery. The efficiency of the first prime editor was comparatively low, but numerous approaches were proposed to solve this problem [5].

There are various cell types in the organism, some dividing and some non-dividing. DNA synthesis is only active in dividing cells, namely, in the S-phase of cell cycle. In adults, most cells are in a non-dividing state. It was previously believed that, unlike HDR-based CRISPR-Cas, the PE system does not rely on cell division for its operation. Additionally, DNA mismatch repair (MMR) was thought to function in non-dividing cells, giving PE a distinct advantage as a genome editing tool for gene therapy [6].

Interestingly, prime editing was found to be dependent on the division status of the target cells. The higher content of actively dividing cells correlates with the higher efficiency of prime editing, as was shown in experiments on myoblasts and myotubes [7]. DNA synthesis mechanism or replication-dependent DNA repair processes can increase the prime editing efficiency in replicating cells. In the case of CRISPR, we can suspect homology-directed repair involvement, but PE does not rely on HDR; therefore, some other mechanism increases PE’s efficiency in dividing cells. DNA mismatch repair is mostly active in dividing cells [8], and heteroduplex resolution in the final stage of the PE mechanism depends on it, so the role of MMR in PE should be analyzed more closely. Here, we aim to analyze the processes which work in favor or against prime editing in dividing cells and to elucidate their contribution to the efficiency of editing.

## 2. Quiescent and Terminally Differentiated Cells

Cellular quiescence is a reversible non-proliferating state, which is classified as a distinct G0 state outside of the cell cycle. Quiescent cells can return to the normal cell cycle under certain physiological conditions [9]. This state is not passive, as it requires the downregulation of proliferation-related genes (such as dNTP synthetic enzymes) and the expression of specific genes and is actively maintained. Examples of quiescent cells are many adult stem cells and progenitor cells. Fibroblasts, lymphocytes, muscle satellite cells, and hepatocytes are also quiescent cells. Cellular quiescence also provides protection against stress and toxicities. Quiescence should not be confused with irreversible cellular senescence.

Terminal differentiation is the process in which cells acquire their specific structural, functional, and biochemical properties. Terminally differentiated cells were described as irreversibly arrested and unable to re-enter the cell cycle. The boundary between terminally differentiated and quiescent cells is not as strict as it was considered previously. Some terminally differentiated cell types, including cultured myotubes, can be brought back into the cell cycle [10]. The transition of fibroblasts to induced pluripotent stem cells is well studied and widely used in practice [11]. In vivo, cell dedifferentiation is a pivotal procedure for animals to deal with injury and promote endogenous tissue repair. In conclusion, terminal differentiation is not as terminal as the name suggests.

Chromatin is the association between DNA and nucleosomes that allows the compaction of DNA in a nucleus. Chromatin’s structure is supposed to influence the genome editing efficiency by interfering with the accessibility of target sites to the Cas9 protein and guide RNA. Transcription factors, such as p53DD and P65, can loosen the dense chromatin structure and make genomic sites more accessible for prime editing [12,13]. Dynamical reorganization of chromatin during the cell cycle was demonstrated in budding yeast [14].

Quiescence is associated with a specific epigenetic state and condensed chromatin structure, which are hypothesized to maintain reversibility of cell cycle arrest [15]. The di- or trimethylation of histone H3 lysine 27 at cell cycle genes, which causes their repression, has been shown to maintain quiescent state [16]. Chromatin’s structure affects the efficiency of MMR, and the inter-relation of chromatin assembly and MMR is not understood conclusively, especially in eukaryotes. The condensed chromatin state may pose a problem when considering DNA accessibility for prime editing machinery, and there were attempts to overcome this obstacle. For example, a prime editing variant with chromatin-modulating peptides (CMP-PE-V1) can open the local chromatin at the target locus to increase the prime editing efficiency up to 2-fold [17].

Some terminally differentiated and, in many cases, quiescent cells are expected to live and function until the death of the organism, so they should have mechanisms of protection against DNA damage. This process should be independent of DNA synthesis, which does not occur in non-dividing cells.

## 3. DNA Mismatch Repair in Prime Editing

In general, genome editing is the process that compromises genome integrity, thereby causing conditions of genotoxic stress. Genotoxic stress response processes involve multiple pathways of DNA damage repair. Five DNA repair mechanisms, which are base excision repair (BER), nucleotide excision repair (NER), mismatch repair (MMR), homologous recombination (HR), and non-homologous end joining (NHEJ), function at various phases of the cell cycle [18]. Mismatch repair is a mechanism which repairs small-scale insertions, deletions, and substitutions during DNA replication and recombination. All of these DNA modifications could be the result of prime editing. Cells that are deficient in MMR have a mutator phenotype, characterized by its high rate of random spontaneous mutations. This condition is associated with some types of cancer [19]. The MMR mechanism involves several proteins (Table 1).

**Figure 2:**
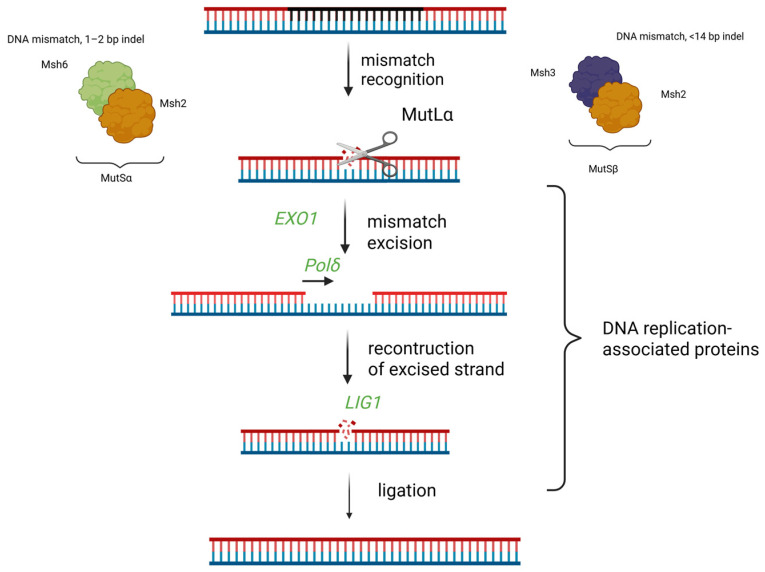
The interplay of DNA mismatch repair and DNA replication mechanisms. The proteins that are involved in both these processes are shown in italics. Mismatch repair starts with two proteins, MutSɑ (Msh2–Msh6 dimer) and MutSβ (Msh2–Msh3 dimer), which recognize base mismatches and indels. The Msh6 subunit recognizes small mutations, such as single-nucleotide mismatches and small insertions/deletions of 1–2 nucleotides. The Msh3 subunit recognizes insertions/deletions of up to 14 nucleotides and was also found to recognize some single-nucleotide mismatches. The Msh2 subunit recruits MutLɑ (PMS2–Mlh1), which incises the nick-containing strand around the heteroduplex. The incised strand is removed by exonuclease 1 (EXO1), while polymerase δ resynthesizes the excised DNA strand on the template of a complementary strand, and ligase I (LIG1) repairs the single strand-break. Created using Biorender.

In mammals, mismatch repair starts with two proteins, MutSɑ (Msh2–Msh6 dimer) and MutSβ (Msh2–Msh3 dimer), which recognize base mismatches and indels that are up to 14 nucleotides in length [20,21]. The Msh6 subunit recognizes small mutations, such as single nucleotide-mismatches and small insertions/deletions of 1–2 nucleotides. The Msh3 subunit recognizes insertions/deletions of up to 14 nucleotides and was also found to recognize some single-nucleotide mismatches [22]. Inactivation of MutSβ was shown to cause a weaker mutator phenotype than inactivation of MutSɑ, which suggests that large indels arise less frequently or are repaired without MMR involvement [23]. The Msh2 subunit does not recognize the mismatches but is necessary for the repair process, because it recruits MutLɑ (PMS2–Mlh1), which incises the nick-containing strand around the heteroduplex [24,25]. The incised strand is removed by EXO1 [26], while polymerase δ resynthesizes the excised DNA strand on the template of the complementary strand, and ligase I (LIG1) repairs the single-strand break [27]. In eukaryotic cells, MMR is tightly coupled with the DNA replication process, and MMR activity is the highest during the S-phase of the cell cycle, when DNA is replicated, while MMR proteins work at replication foci, being activated there specifically by Proliferating Cell Nuclear Antigen (PCNA) [28]. The expression of PCNA, FEN1, and LIG1 is downregulated in quiescent cells [8]. FEN1 and LIG1 are involved in DNA mismatch repair, base excision repair, and ribonucleotide excision repair. The role of DNA replication mechanism in mismatch repair is presented in Figure 2.

The MMR efficiency was found to decrease depending on the mismatches in the following order: G:T = G:G > C:A = A:A = A:G >> G:A = C:C = T:T [29]. A low efficiency for C:C mismatches was confirmed both in *Escherichia coli* and human cells [30,31,32]. This effect was used to improve the efficiency of prime editing [33], as G:C-to-C:G prime editing creates a C:C mismatch as an intermediate. This finding supports the negative role of MMR in prime editing.

An important question is the mechanism of recognition of the strand that is to be excised. In prokaryotes, the MMR machinery recognizes the absence of methylation of newly synthesized strands [30], but this mechanism does not function in eukaryotes. In eukaryotes, different mechanisms for targeting MMR to either a leading or lagging strand during replication were proposed. The role of the asymmetrically loaded PCNA-DNA complex in strand recognition is known in prokaryotes and was proposed for eukaryotes [34]. The interaction between PCNA and reverse transcriptase is not described. As PCNA is downregulated in non-dividing cells, its role in strand recognition in MMR without replication should be quite small. In the case of the lagging strand, multiple 5′-termini of Okazaki fragments might be the signal for recruiting MMR machinery [35], and in the case of the leading strand, it might be the nascent DNA 3′-termini. Therefore, nicks are the signal for MMR, even outside of the replication context. The role of different strand recognition factors for MMR in different cell states requires further investigation.

Chen et al. proposed [33] that the PE DNA heteroduplex, which is formed by hybridization of the newly synthesized 3′ DNA flap carrying the edit to non-edited genomic DNA, undergoes MMR. MMR resolves DNA heteroduplexes by selectively replacing nicked DNA strands [36]; therefore, the PE3 editor specifically relies on nicking the non-edited strand to direct the MMR system to it. Here, MMR in fact works in favor of editing. On the other hand, the high activity of MMR was shown to decrease PE efficiency with an increase in erroneous indel byproducts [33]. Interestingly, Chen et al. found that the inhibition of MMR enhances PE efficiency to a lesser extent in PE3 than in PE2 (2.0-fold and 7.7-fold, respectively).

Ferreira da Silva et al. [37] tested the effect of MMR inhibition on PE efficiency and found that MMR activity is a negative factor for PE. MutLɑ, Msh2, Msh3, and EXO1 inhibition was found to increase PE efficiency, but the design of the experiment did not allow for the testing of the efficiency of Msh6 inhibition, as it does not recognize 5 bp deletion. In contrast, the knock-down of LIG1 reduced the frequency of intended editing, which was consistent with its previously proposed role in nick ligation [1]. The activity of LIG1 was found to be increased in dividing cells [38] but nonetheless present in non-dividing cells at some base level, because LIG1 is involved in multiple DNA damage repair pathways, some of which are active both in dividing and non-dividing cells.

Prime editors were developed and improved gradually. PE1 consists of an nCas9 nickase that is linked to the wild-type Moloney murine leukemia virus reverse transcriptase. PE2 features an engineered pentamutant MMLV RT, which enhances the editing efficiency by approximately threefold. PE3 builds upon the PE2 fusion protein, incorporating pegRNA and an additional gRNA that targets the non-edited strand for nicking, thereby further improving the editing efficiency. The PE4 and PE5 prime editing systems were created by Chen et al. [33]. They involve derivatives of PE2 and PE3. These systems utilize the transient expression of an engineered dominant negative protein, Mlh1 Δ754–756 (MLH1dn), which partially inhibits mismatch repair and enhances the efficiency of substitution, small insertions, and small deletions in prime editing compared to the PE2 and PE3 systems. Additionally, they improve the edit/indel ratios in MMR-proficient cell types. Notably, the introduction of extra silent mutations in the edited region has been suggested as a strategy to diminish the MMR machinery’s recognition of heteroduplexes, thereby reducing its activity [39]. This may account for the increased efficiency of multiple-nucleotide substitution repair templates in plants, which is particularly evident in the case of PE3 [40]. As PE3 relies on MMR in its degradation of non-edited strands, the improvement in efficiency between PE3 and PE5 is less significant than between PE2 and PE4.

Human embryonic kidney 293 cells (HEK293T), which are notable for their fast growth and widely used in research, demonstrate low MMR activity. Because of this, these cells can provide a valuable insight into the role of MMR in prime editing. Interestingly, HEK293T demonstrated an increased PE efficiency [33]. This finding implies that the less MMR-proficient the cells are, the more efficient they are at prime editing. For example, pluripotent stem cells, which are characterized by high MMR levels [41], should be a poor target for PE-based genome editing. We might suggest a model where activities of MMR (particularly MutLɑ) and ligase 1 are the opposing factors: the high nicking activity leads to MMR removing the edit, and the high ligating activity leads to edit incorporation. Therefore, the most efficient prime editing is to be expected in cells demonstrating low MMR activity and high ligase 1 activity, and cancer cells should be particularly susceptible to prime editing, as MMR was found to be inactive in many cancer phenotypes [19], while ligase 1 activity was elevated [42]. This opens prospects both for the treatment of pre-existing cancer cells, as well as for creating new models for oncological research using cells with mutator phenotypes.

Mismatch repair is able to produce mutations too. MMR machinery was found to participate in somatic hypermutation, a mutagenic process in B lymphocytes that generates immunoglobulin diversity. This type of MMR-dependent mutagenesis has been attributed to a non-canonical MMR (ncMMR) mechanism which is activated in a variety of cell types and is independent of DNA replication [43]. First, Activation-Induced Cytidine Deaminase (AID) targets single-stranded DNA of rearranged Ig genes and converts deoxycytidines to deoxyuracils. AID has a preference for the WRCY motif (W = A/T, R = A/G, and Y = C/T) [44]. Then, one of the following occurs: (1) replication generates transitions at C/G; (2) uracil-DNA glycosylase-2 (UNG2)-dependent translesion synthesis or ncMMR generates transversions at C/G; or (3) ncMMR or UNG2 and PCNA ubiquitination generate mutations at A/T. The trigger of ncMMR activation is Msh2/Msh6 recognizing the U:G mismatch, generated by AID. Outside of the DNA replication context, ncMMR employs error-prone POLH polymerase, which generates all types of mutations [45]. Therefore, the activity of MMR is not linked to DNA replication, but outside of a DNA replication context, it does not employ high-fidelity replicative polymerase δ, making it susceptible to mutagenesis.

In general, MMR and PE might be seen as competing processes, partially employing the same cellular mechanisms, as both of them are dependent on DNA replication conditions, but with opposing outcomes. Therefore, (1) PE and MMR are active under similar conditions, and the (2) inhibition of MMR should promote PE given the similar cell state. But MMR is able to introduce new mutations, while repairing mismatch, especially when it employs error-prone polymerases. It would be advantageous to use MMR to incorporate the desired edit by targeting non-edited strands or by employing low-fidelity polymerase. The former should be addressed with PE3-type editing, where a non-edited strand is nicked to hinder strand recognition for the MMR machinery, as a nick should be the main factor for strand discrimination. The latter may be possible in non-dividing cells outside of the DNA replication context.

## 4. Reverse Transcription in Non-Dividing Cells, dNTP Concentration, and Prime Editing

Reverse transcription is less effective in quiescent cells, as it has a lower rate and terminates prematurely, which could be a mechanism of anti-viral defense [46]. Non-dividing cells contain low levels of dNTPs compared to dividing cells, resulting in an extremely low dNTP/rNTP ratio, and this is the reason for the inefficiency of reverse transcription in non-dividing cells [47]. Lentiviruses, such as HIV-1, infect both dividing and non-dividing cells, whereas retroviruses such as Moloney Murine Leukemia Virus only infect actively dividing cells. The affinity for dNTPs of the MMLV reverse transcriptase that is used for prime editing is 7- to 122-fold lower than HIV-1 reverse transcriptase [48]. In addition, increasing the MMLV RT affinity for dNTPs with the V223M mutation enhances the prime editing efficiency in quiescent hematopoietic stem and progenitor cells (HSPCs) and resting primary T cells up to 1.2-fold [39].

The low dNTP/rNTP ratio is caused by SAMHD1 protein activity [49,50,51] and by the repression of the enzymatic pathways that are involved in dNTP synthesis. SAMHD1 hydrolyzes intracellular dNTPs, inhibiting reverse transcription with an explicitly anti-retroviral function. Quiescent and terminally differentiated cells demonstrate an increase in SAMHD1 expression [52]. The addition of deoxynucleosides to the culture medium was found to accelerate HIV-1 reverse transcription in infected monocyte-derived macrophages, indicating that the intracellular dNTP pool is limited in these cells [50]. Therefore, we might suggest the addition of dNTP to the cultural medium for prime editing of non-dividing cells in vitro. This approach would be limited, since dNTPs normally do not penetrate intact plasma membranes. On the other hand, deoxynucleosides (dNs) can be transported inside the cells through equilibrative nucleoside transporters (ENTs) and converted to dNTPs via the dNs salvage pathway. Indeed, supplementing dNs in the culture media has been shown to enhance lentiviral transduction in resting primary T cells [53] or quiescent HSPCs [54]. A 2-fold increase in PE efficiency was shown upon dN supplementation [39].

Alternatively, SAMHD1 knock-out could be proposed specifically for non-dividing cells, because in dividing cells, siRNA silencing of SAMHD1 stops cells in the G1 (pre-synthesis) phase of the cell cycle, blocking DNA replication altogether [55]. Recently, a treatment was proposed (Qi et al., 2023 [56]) with the SAMHD1 inhibitor and cephalosporin C zinc salt, and a significant improvement of prime editing efficiency (4-fold on average) was observed. The delivery of the viral protein Vpx, which guides SAMHD1 for degradation, was also applied [52]. The effects of SAMHD1 knock-out in quiescent cells were not extensively described. dNTP supplementation and SAMHD1 degradation with Vpx can be combined [39].

## 5. Prime Editor Delivery in Dividing and Non-Dividing Cells

Non-dividing cells pose particular problems for the delivery of DNA-editing machinery. RNA or plasmid delivery both require the active translation of the genome editor, which could be limiting in non-dividing cells that are in metabolic dormancy [56]. On the other hand, many types of non-dividing cells are metabolically active, such as neurons, hepatocytes, and muscle fibers. DNA translocation to the nucleus is not effective in quiescent cells [57], as it mostly occurs through nucleus membrane breaks during mitosis [58]. Lentiviral and AAV delivery is, nevertheless, promising in quiescent cells as well [59,60,61,62].

The challenge of delivering prime editors using adeno-associated virus (AAV) stems from the size constraints of the AAV genome, which is approximately 4.7 kilobases (kb), while the size of prime editor is around 6.3 kb; therefore, it cannot be packaged into a single AAV vector. To overcome this limitation, a dual-AAV delivery system was developed [62,63,64]. In the dual-AAV approach, the necessary components for prime editing are split between two separate AAV vectors. This allows for the delivery of the larger prime editor construct by using two smaller AAV vectors that each fit within the packaging constraints. One AAV vector can carry the prime editing guide RNA (pegRNA) and the associated nickase, while the second vector can deliver the reverse transcriptase. Upon the co-infection of target cells with both AAV vectors, they can recombine within the cell to form the complete prime editing system, enabling efficient genome editing.

It is important to further explore the prospects of using prime editing in vivo in non-dividing cells. As previously mentioned, the efficiency of prime editing in these cells is anticipated to be lower than that in dividing cells. Additionally, non-dividing cells face another challenge: the low efficiency of delivering genetic constructs using certain methods.

While almost all delivery methods can target non-dividing cells —such as adeno-associated, adenoviral, and lentiviral vectors [65] or lipid nanoparticles carrying transgene mRNA [66]—non-viral DNA delivery methods (e.g., polyplexes [67]) must first enter the nucleus during cell division, which means that they are less effective in non-dividing cells.

Certain types of non-dividing cells, such as neurons, have been shown to re-enter the cell cycle—at least up to the S phase—before typically undergoing cell death [68]. As noted earlier, the activity of key factors involved in prime editing is maximal during the S phase [8,38]. Additionally, there are both in vitro and in vivo strategies for activating the cell cycle, as demonstrated in corneal endothelial cells [69]. Combining prime editing with cell cycle re-entry presents a promising and potentially effective approach for genome editing in non-dividing cells.

## 6. RNAse H in Prime Editing

MMLV reverse transcriptase contains the ribonuclease H domain (RNase H). RNase H hydrolyzes the RNA strand in an RNA/DNA hybrid [70], which was found to reduce PE efficiency. RNase H involvement in replication-associated MMR was proposed, and it was found to nick DNA with misincorporated ribonucleotides, which arise during DNA replication. The misincorporation of ribonucleotides during reverse transcription in the case of a low dNTP/rNTP ratio is possible; for example, HIV 1 RT was found to incorporate rNTPs into the nascent DNA strand under a low dNTP/rNTP ratio, such as in quiescent cells [71]. Such mispairing plays a role in distinguishing between templates and newly synthesized DNA strands for the purposes of mismatch repair. Particularly, RNase H’s interaction with misincorporated ribonucleotides might play a role in strand discrimination for mismatch repair. Therefore, the removal of the RNase H domain should improve the efficiency of PE both in general and in quiescent cells, specifically at the cost of the possible misincorporation of ribonucleotides, which is not particularly dangerous [72]. Continuous evolution and protein engineering enabled the development of compact and efficient prime editors, including PE6d, which harbors an RNAse H-truncated MMLV reverse transcriptase and has shown increased efficiency [60].

The native cellular ribonucleases H1 and H2 have different cell-cycle-dependent behaviors: H1 is expressed independently of the cell cycle phase, and H2 is specific for G2/M phases. The efficiency of post-replication mismatch repair in Saccharomyces cerevisiae is reduced by inactivating RNase H2, and the genomic instability is increased [73,74]. In humans, RNAse H2 dysfunction is linked with Aicardi–Goutieres syndrome [72]. Non-dividing cells should express RNase H1, and we speculate that RNase H1 inhibition should increase PE efficiency in non-dividing cells.

## 7. Conclusions

**Figure 3:**
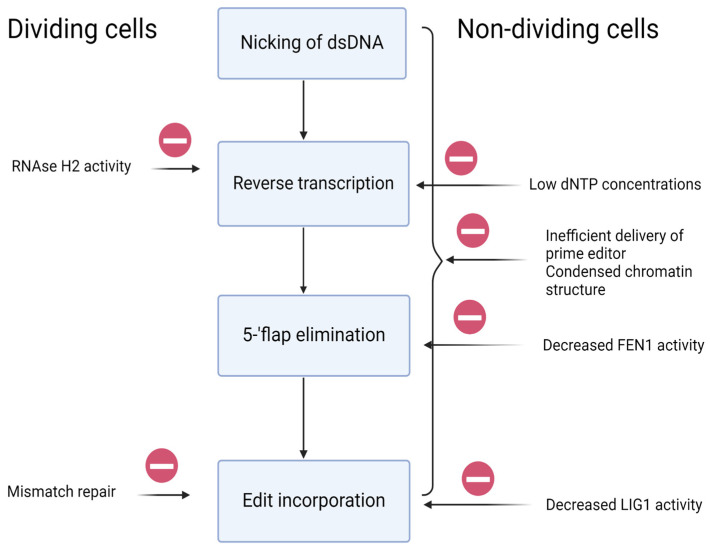
Prime editing in dividing and non-dividing cells. The process of prime editing is broken into 4 steps (nicking of dsDNA, reverse transcription, 5′-flap elimination, and edit incorporation). The factors inhibiting each step in dividing and non-dividing cells are marked with minus signs. Created using Biorender.

The main factors that work in favor of prime editing in dividing cells are the high level of dNTPs, which are necessary for DNA replication but also for reverse transcription, and the elevated expression of proteins that are involved in DNA replication and subsequent DNA repair, which are also a necessary part of the prime editing process, namely FEN1 and LIG1. There are factors which work against the efficiency of prime editing in dividing cells, primarily DNA mismatch repair, which removes the intended edits and incorporates indels. On the other hand, the incorporation of an edit might also rely on the mismatch repair mechanism. MMR inhibition was found to significantly improve the efficiency of prime editing, suggesting that its net effect on prime editing is negative. The repair system features described above undoubtedly impact the overall efficiency of genome editing using prime editing. The different factors affecting PE efficiency are summarized in Figure 3. While the efficiency of editing with PE is not directly tied to the cell cycle phase and cell division, a decrease in efficiency can be anticipated when it is used in non-dividing cells. Nevertheless, we anticipate that the editing efficiency with PE will be higher in quiescent cells compared to HDR-based CRISPR-Cas, which will enable the development of more effective approaches for gene therapy in human diseases.

## Figures and Tables

**Table 1:** Mammalian proteins involved in mismatch repair.

Protein Name	Function
Msh2 (MutS protein homolog 2)	MutLɑ (PMS2–MLH1) recruitment
Msh3 (MutS protein homolog 3)	1–14 bp indel recognition, some base pair mismatch recognition
Msh6 (MutS protein homolog 6)	Mismatch and 1–2 bp indel recognition
MutLɑ (PMS2–Mlh1) (Mismatch repair endonuclease PMS2 (postmeiotic segregation increased 2)—MutL protein homolog 1)	Incision of nick-containing strand
PCNA (Proliferating cell nuclear antigen)	Activation of MutLɑ in replication context
EXO1 (Exonuclease 1)	Removal of incised strand
Polymerase δ	Resynthesis of removed strand
LIG1 (Ligase 1)	Ligation of newly synthesized sequence
